# Potential spillover effect of eco-directed pharmaceutical disposal on pro-environmental disinfectant use among Chinese residents

**DOI:** 10.3389/fpubh.2025.1707924

**Published:** 2025-11-26

**Authors:** Xintong Chen, Yongxin Tong, Jun Wang

**Affiliations:** Institute of Pharmaceutical Innovation, Hubei Province Key Laboratory of Occupational Hazard Identification and Control, School of Medicine, Wuhan University of Science and Technology, Wuhan, China

**Keywords:** disinfection, ecopharmacovigilance, emerging contaminants, environmental spillover, knowledge-attitude-practice, pharmaceutical emerging contaminants

## Abstract

**Background:**

The growing use of disinfectants has raised ecological concerns regarding disinfectants as emerging environmental contaminants. Given the similarities in environmental fate and discharge behavior between pharmaceuticals and disinfectants, ecopharmacovigilance (EPV) as a framework emphasizing eco-directed pharmaceutical use and disposal was previously proposed as a potential strategy to mitigate environmental emissions of disinfectants. This study investigated the likelihood of spillover between eco-directed pharmaceutical disposal and pro-environmental disinfectant use across knowledge, attitude, and practice (KAP) dimensions, and examine whether perceived similarity between pharmaceuticals and disinfectants mediated these spillover effects.

**Methods:**

Using a sample of 1,002 Chinese residents, questionnaire-based KAP survey data were analyzed using regression analysis for spillover effect test. A bias-corrected non-parametric percentile Bootstrap test was employed to examine mediation effects.

**Results:**

A one-unit increase in eco-directed pharmaceutical disposal improved the residents’ pro-environmental disinfectant use by 0.648, 0.782, and 0.791 units in KAP dimensions (*p* = 0.000), respectively, under the control of covariates. Similarity recognition exerted a significant mediating effect on the relationship between eco-directed pharmaceutical disposal and pro-environmental disinfectant use, with the effect size values of 0.168 (95% confidence interval [CI]: 0.130, 0.206), 0.107 (95% CI: 0.072, 0.142), and 0.079 (95% CI: 0.029, 0.128) across KAP dimensions, respectively.

**Conclusion:**

Eco-directed pharmaceutical disposal might positively spill over to pro-environmental disinfectant use partially through indirect path of the perceived similarity, which further supports the feasibility of adopting EPV, a framework originally designed for pharmaceutical environmental risk management, for the remediation of disinfectant emerging contaminants.

## 1 Introduction

Disinfectants are widely used in healthcare facilities, households, public places, and livestock farms to inactivate or eliminate pathogenic microorganisms, thereby reducing the risks of infectious diseases (1–3). Especially in the home-based settings, diverse chemical disinfectants represented by alcohols, hypochlorite and hypochlorous acid, phenolics, guanidine bactericides, quaternary ammonia are widely used by the general public, in the forms of disinfectant sprays, hand sanitizers, wipes, soaps, detergents, toothpaste, mouthwash, deodorant, shampoo, and other personal care products (2–4). Over the past 5 years, the importance of disinfectant use has been increasingly highlighted because of the frequent emergence and transmission of infectious diseases, such as coronavirus disease 2019 (COVID-19) and influenza type A (4, 5). Data from Canada indicated that during the COVID-19 pandemic, the sales indices of disinfectants and hand sanitizers consistently remained close to 150 and 300% of the 2019 weekly average, respectively (6). When Chinese residents self-evaluated the impact of the COVID-19 outbreak on their disinfectant use levels in 2020, over 96.0% of the respondents reported that their overall disinfectant use had more than doubled due to the outbreak, and 26.5% thought that their use levels of hand sanitization products had increased by more than 10 times (4). Even after the COVID-19 pandemic, a considerable proportion of residents tend to often or always use disinfectant products in their daily life, perhaps because of habit formation and enhanced public hygiene awareness (7, 8). Importantly, such growing use of disinfectants has raised substantial ecological concerns associated with their excessive release into the environment, subsequent diffusion, and accumulation in ecosystems (1, 4, 9–11).

Currently, disinfectants in the environment have been recognized as a representative category of emerging contaminants, in view of their continuous environmental discharge without targeted regulatory oversight, ubiquitous occurrence in environmental matrices, and their known potential to induce antimicrobial resistance and ecotoxicity in non-target organisms even at environmentally relevant concentrations (1, 4, 10, 12). A recent study in New York State, USA (9) found that quaternary ammonium compounds (QACs), common household disinfectants, are widely detected as contaminants in surface and drinking waters. The study estimated annual average QAC discharge into the Atlantic Ocean via the Hudson River at 21,000 kg, with risk quotients reaching up to 5,260 in some collected samples. During the COVID-19 pandemic, chlorination disinfection by-products were frequently detected in surface water and tap water samples collected in Wuhan, China, with maximum concentrations reaching 97.4 and 56.0 μg/L, respectively (13). In an evaluation of 135 pharmaceuticals and personal care products (PPCPs) at seven representative Canadian wastewater treatment plants conducted in 2022 (14), triclosan as a household-derived disinfectant was detected in treated biosolids at a median concentration of 1,545 ng/g, ranking third highest among all tested PPCP. Para-chloro-meta-xylenol (PCMX) is another disinfectant widely used in consumer product such as hand sanitizers and household cleaners. Au et al. (15) reported a large-scale and persistent PCMX pollution in the river and coastal water environments in Hong Kong, with residual concentrations ranging from 0.1 to 10.6 μg/L. Chemically enhanced primary treatment failed to effectively remove PCMX in wastewater, and PCMX remains relatively stable even after being released in the seawater environment.

Furthermore, growing evidence has demonstrated that disinfectants at environmentally relevant concentrations could induce multiple toxic effects on non-target aquatic organisms (10, 11, 16–19). Triclosan and triclocarban have been well established as prototypical endocrine-disrupting pollutants in aquatic environments, where they interfere with hormonal pathways in organisms ranging from invertebrates to fish (16, 17). Liu et al. (19) explored the developmental toxic effects of five common chemical disinfectants, triclosan, triclocarban, benzalkonium chloride, benzethonium chloride, and PCMX, on zebrafish embryos (*Danio rerio*) at exposure concentrations of 0.4–40 μg/L. Results showed that all tested disinfectants except PCMX induced significant teratogenic toxicities. Another study showed the environmental risks posed by QACs as emerging contaminants on three model organisms, *Cyprinus carpio, D. rerio,* and *Xenopus laevis*, based on embryotoxicity analysis (18). A range of adverse effects associated with polyhexamethylene guanidine, a guanidine disinfectant, on the model alga *Chlorella vulgaris*, from slight disturbances at low concentrations to significant growth inhibition at higher levels, have been recently reported by Zhang et al. (11), suggesting a hormesis-like response. More importantly, the inherent biocidal activity of these disinfectant compounds raised urgent concerns about their antimicrobial resistance properties in the environment (12, 20, 21). To date, multiple classes of disinfectants, such as peracetic acid (22), QACs (21, 23), triclosan (24), chlorine-containing disinfectants (25), have been reported to exert selective pressure on environmental microorganisms, and promote the development of antimicrobial resistance at environmentally relevant concentrations, thereby creating a critical and growing challenge to public health.

Nevertheless, no management strategy is available to address the environmental discharge of disinfectant emerging contaminants (DECs). Previous studies (4, 10, 12) proposed that, given multiple similarities of DECs and pharmaceutical emerging contaminants (PECs) in contaminant classification, environmental fate, risk profiles, and anthropogenic sources, etc., ecopharmacovigilance (EPV) that originally developed as a pharmaceutical risk management framework to control environmental hazards from PECs, could be adopted to mitigate DEC-related risks. The implementation of EPV relies on targeted drug administration protocols, such as the use of green alternatives, sustainable prescribing, eco-directed disposal of expired or unused medication (26–30). Extending this EPV framework, upstream source-control measures for DECs have been proposed, such as adopting green disinfectant alternatives, reducing disinfectant misuse/overuse, and implementing eco-friendly disposal of unused disinfectants (4, 10, 12). However, empirical evidence to validate the feasibility of this EPV-based remediation strategy for DECs remains limited.

Positive spillover effects of environmentally-responsible behaviors from one setting to another have been increasingly recognized, because when they occur, sustainable interventions targeting specific pro-environmental behavior (PEB) would become more effective and efficient by also increasing sustainability of subsequent PEBs (31–38). Notably, the perceived similarity between settings of the initial and subsequent behaviors can facilitate spillover (31–33, 35–38). Here, we speculated that eco-directed pharmaceutical disposal, i.e., participation in drug take-back system, which is currently the most accessible EPV practice in China (30, 39, 40), could exhibit positive spillover to pro-environmental disinfectant use. This study formulated the following research hypotheses:

*H1*: Understanding about eco-directed pharmaceutical disposal has a significantly positive impact on knowledge of pro-environmental disinfectant use.

*H2*: Attitude toward eco-directed pharmaceutical disposal has a significantly positive impact on opinions regarding pro-environmental disinfectant use.

*H3*: Eco-directed pharmaceutical disposal behavior has a significantly positive impact on PEB related to disinfectant use.

*H4*: Perceived similarity between PECs and DECs positively mediates the relationship between eco-directed pharmaceutical disposal and pro-environmental disinfectant use.

The above hypotheses were tested based on knowledge-attitude-practice (KAP) survey data from Chinese residents. If true, EPV practices as sustainable interventions targeting eco-directed pharmaceutical use and disposal behaviors could contribute to the upstream control of anthropogenic environmental emissions of disinfectants.

## 2 Methods

### 2.1 Study design and participants

A questionnaire-based cross-sectional survey was conducted among a convenience sample of Chinese residents from March to July, 2024. Survey-informed consent was administered to all participants along with the questionnaire, and was received from those who completed the questionnaire.

Inclusion criteria of respondents were (1) Chinese adults aged over 18 years, (2) willingness to participate voluntarily, and (3) compliance with completing the electronic questionnaire independently. Initial respondents were contacted via personal connections of the co-authors, then snowball sampling approach was employed to expand recruitment among additional respondents.

### 2.2 Questionnaire

An initial draft of the questionnaire was developed based on information from the relevant literature (4, 9, 11), and pretested among a convenience sample of 20 residents, who were excluded from the final survey. Content validity, item relevance, clarity, and conciseness of the questionnaire were assessed by two senior researchers engaged in pharmacy administration and sanitation management. Based on feedback from pretest participants and the senior researchers, minor revisions were made to finalize the survey questionnaire.

The final version of the questionnaire (Supplementary material) comprised four sections with 31 total items. The first section on socio-demographic information encompassed 5 items capturing respondents’ gender, age, residence, education level, and professional background. The second section included 10 items scored on a 5-point Likert scale ranging from 1 (strongly disagree) to 5 (strongly agree), which were organized into three dimensions, knowledge (K), attitude (A), and practice (P), associated with eco-directed pharmaceutical disposal. In the knowledge dimension, three questions were designed to assess the respondents’ understanding regarding the final entrance and environmental risks of pharmaceuticals in the environment, and the relationship between improper disposal of unused/expired drugs and pharmaceutical pollution. The attitude dimension included three questions to measure opinions about the adverse effects of PECs on human health and the ecosystem, the importance of source control for pharmaceutical pollution, and the willingness to modify behaviors for pharmaceutical pollution control. Four practice questions were designed to evaluate compliance with eco-directed pharmaceutical disposal practices. Consistent with the second section, another 10 parallel Likert-scored questions were included in the third section to assess KAP related to pro-environmental disinfectant use. Given the lack of a well-accepted behavioral framework for pro-environmental disinfectant use, the practice dimension specifically focused on the green alternative practice using environmentally friendly disinfectants highlighted in previous studies (1, 12, 41). In the last section of the questionnaire, the respondents were asked about 6 question items adapted from the previous fast survey (12) to assess respondents’ perceptions of similarities between PECs and DECs. Either Cronbach’s *α* value or the Kaiser-Meyer-Olkin (KMO) value of the final questionnaire exceeded 0.700.

The questionnaire was originally developed in English and subsequently translated into Chinese for distribution among the Chinese population. The final questionnaire was imported into the Questionnaire Star mini-program to generate an anonymous survey link, which was shared via WeChat, the most widely used and popular social media platform in China.

### 2.3 Statistical analysis

Collected survey data were entered into SPSS 26.0 for descriptive statistical analysis. The correlations of the studied variables were analyzed by Pearson correlation analysis. Regression analysis was used to test the spillover effect test. The mediation effect analysis was conducted through the bias-corrected non-parametric percentile Bootstrap test (34, 42). Specifically, the PROCESS macro program in SPSS 26.0 and model 4 (the simple mediation model) of Hayes were used to test the H4. Group differences were analyzed using an independent samples t-test for comparisons between two groups and one-way analysis of variance (ANOVA) for comparisons among three or more groups. When the *p* value was below 0.05 or 0.01, the differences would be considered statistically significant.

## 3 Results

### 3.1 Socio-demographic characteristics

By the end of study period, a total of 1,167 Chinese residents responded to the questionnaires. After excluding invalid questionnaires that were uncompleted (*n* = 45), answered by respondents who self-reported being under 18 years of age (*n* = 39), or provided with identical responses to all question items (*n* = 81), there were 1,002 valid questionnaires included for final analysis, resulting in a response validity rate of 85.86%. Socio-demographic characteristics of the valid sample are present in Table 1.

**Table 1:** Descriptive statistics for the Chinese residents’ knowledge, attitude, and practice (KAP) scores regarding eco-directed pharmaceutical disposal or pro-environmental disinfectant use, and their recognition of PEC-DEC similarity.

Participant attribute	*n* (%)	Eco-directed pharmaceutical disposal						Pro-environmental disinfectant use						Perceived similarity scores ^c^	
		Knowledge ^a^		Attitude ^a^		Practice ^b^		Knowledge ^a^		Attitude ^a^		Practice ^b^			
		Average score ± SD	*p* value	Average score ± SD	*p* value	Average score± SD	*p* value	Average score ± SD	*p* value	Average score ± SD	*p* value	Average score ± SD	*p* value	Average score ± SD	*p* value
Total scores	1,002 (100)	4.12 ± 0.72		4.13 ± 0.68		3.83 ± 0.68		4.21 ± 0.68		4.11 ± 0.68		3.47 ± 0.87		4.34 ± 0.62	
Gender															
Male	503 (50.2)	4.08 ± 0.71	0.041*	4.07 ± 0.70	0.008**	3.80 ± 0.71	0.099	4.17 ± 0.65	0.039*	4.05 ± 0.72	0.002**	3.53 ± 0.89	0.007**	4.28 ± 0.65	0.000**
Female	499 (49.8)	4.16 ± 0.72		4.18 ± 0.66		3.86 ± 0.68		4.25 ± 0.70		4.17 ± 0.64		3.40 ± 0.83		4.41 ± 0.58	
Age															
18–20 years	84 (8.4)	4.06 ± 0.68	0.005**	3.98 ± 0.69	0.014**	3.56 ± 0.68	0.000**	4.11 ± 0.68	0.011**	4.03 ± 0.68	0.014**	3.24 ± 0.85	0.527	4.22 ± 0.67	0.008**
21–40 years	532 (53.1)	4.15 ± 0.70		4.14 ± 0.68		3.77 ± 0.71		4.22 ± 0.68		4.12 ± 0.69		3.50 ± 0.84		4.28 ± 0.64	
41–65 years	290 (28.9)	4.18 ± 0.73		4.24 ± 0.65		3.99 ± 0.58		4.27 ± 0.67		4.19 ± 0.66		3.60 ± 0.84		4.49 ± 0.57	
> 65 years	96 (9.6)	3.84 ± 0.73		3.84 ± 0.72		3.90 ± 0.73		4.07 ± 0.72		3.90 ± 0.68		3.10 ± 0.95		4.33 ± 0.59	
Place of residence															
Municipalities or provincial capital cities	586 (58.5)	4.24 ± 0.68	0.000**	4.22 ± 0.63	0.000**	3.93 ± 0.66	0.000**	4.33 ± 0.63	0.000**	4.19 ± 0.63	0.000**	3.54 ± 0.80	0.000**	4.41 ± 0.62	0.000**
Other cities	191 (19.1)	3.93 ± 0.70		3.90 ± 0.73		3.72 ± 0.73		4.04 ± 0.72		3.91 ± 0.75		3.24 ± 0.97		4.30 ± 0.65	
Villages and towns	225 (22.5)	3.97 ± 0.73		4.10 ± 0.70		3.65 ± 0.66		4.05 ± 0.70		4.07 ± 0.68		3.52 ± 0.86		4.21 ± 0.59	
Education level															
High school or below	215 (21.5)	3.66 ± 0.76	0.000**	3.71 ± 0.80	0.000**	3.55 ± 0.79	0.000**	3.79 ± 0.76	0.000**	3.72 ± 0.81	0.000**	3.07 ± 1.09	0.000**	4.01 ± 0.69	0.000**
Junior college	243 (24.3)	4.04 ± 0.69		4.10 ± 0.65		3.88 ± 0.61		4.18 ± 0.62		4.07 ± 0.64		3.48 ± 0.87		4.38 ± 0.62	
Under graduate	336 (33.5)	4.29 ± 0.61		4.26 ± 0.59		3.88 ± 0.64		4.35 ± 0.59		4.26 ± 0.58		3.63 ± 0.72		4.44 ± 0.56	
Post graduate	208 (20.8)	4.41 ± 0.59		4.36 ± 0.53		3.99 ± 0.67		4.45 ± 0.61		4.31 ± 0.55		3.60 ± 0.68		4.49 ± 0.54	
Healthcare professional background															
Yes	279 (27.8)	4.50 ± 0.63	0.000**	4.43 ± 0.55	0.000**	4.06 ± 0.64	0.000**	4.54 ± 0.60	0.000**	4.38 ± 0.57	0.000**	3.52 ± 0.79	0.138	4.56 ± 0.53	0.000**
No	723 (72.2)	3.97 ± 0.69		4.01 ± 0.69		3.74 ± 0.68		4.08 ± 0.67		4.00 ± 0.69		3.45 ± 0.90		4.26 ± 0.64	

*p < 0.05, **p < 0.01.

^a^Shown as the average score ± SD of three knowledge or attitude questions, each of which was framed into a 5-point Likert-scale format.

^b^Shown as the average score ± SD of four practice questions, each of which was framed into a 5-point Likert-scale format.

^c^Shown as the average score ± SD of six questions in the fourth section of the questionnaire, each of which was framed into a 5-point Likert-scale format.

Among the 1,002 responding Chinese residents, males and females each accounted for approximately half; 84 (8.4%) respondents aged 18 to 20, 532 (53.1%) aged between 21 and 40 years old, 290 (28.9%) aged between 41 and 65 years old, and 96 (9.6%) were over the age of 65. Most of the respondents lived in cities, among which 586 (58.5%) lived in municipalities or provincial capital cities, and 191 (19.1%) lived in other cities. Educational levels of nearly half (45.7%) of respondents were below undergraduate, while about one third (33.5%) of respondents had completed undergraduate education, and 208 (20.8%) held a postgraduate degree. The majority of responding residents (72.2%) did not have a healthcare professional background.

### 3.2 Descriptive findings

As presented in Table 1, the average scores for eco-directed pharmaceutical disposal across the KAP dimensions were as follows: 4.12 (knowledge, 3 items), 4.13 (attitude, 3 items), and 3.83 (practice, 4 items) (all scored on a 5-point Likert scale). For pro-environmental disinfectant use, the corresponding mean KAP scores were 4.21 (knowledge), 4.11 (attitude), and 3.47 (practice) (also 5-point Likert scale). Additionally, the average score for the 6-item perceived similarity scale assessing respondents’ perceptions of similarity between PECs and DECs was 4.34 ± 0.62 (5-point scale).

Group comparison showed that all the considered socio-demographic characteristics exerted significant effects on not only the KAP of eco-directed pharmaceutical disposal and pro-environmental disinfectant use, but also the perceived similarity scores (*p* < 0.05; *p* < 0.01). Specifically, females knew better than males (*p* < 0.05), and held more positive attitudes toward eco-directed pharmaceutical disposal and pro-environmental disinfectant use, and exhibited greater recognition of PEC-DEC similarity (all *p* < 0.01). However, males were more likely to engage in pro-environmental disinfectant use behaviors (*p* < 0.01). Participants aged 41–65 years, residing in municipalities or provincial capitals, with higher educational attainment, or with a healthcare professional background achieved significantly higher scores across all KAP dimensions of eco-directed pharmaceutical disposal and pro-environmental disinfectant use, as well as perceived similarity scores, relative to their respective counterparts (*p* < 0.05; *p* < 0.01).

Table 2 displays the Pearson’s correlation coefficients for all the studied variables. Results showed that the KAP dimensions of eco-directed pharmaceutical disposal, the KAP dimensions of pro-environmental disinfectant use, and perceived between PECs and DECs were all significantly and positively correlated (*p* < 0.01). In particular, the knowledge about eco-directed pharmaceutical disposal was significantly correlated with knowledge levels regarding pro-environmental disinfectant use (*r* = 0.722); in the attitude dimensions, the residents’ opinions toward eco-directed pharmaceutical disposal were significantly correlated with those regarding pro-environmental disinfectant use, with a correlation coefficient of 0.807; while the correlation coefficient between eco-directed pharmaceutical disposal practice and pro-environmental disinfectant use behaviors was 0.598.

**Table 2:** Pearson’s correlation coefficients among KAP dimensions of eco-directed pharmaceutical disposal and pro-environmental disinfectant use, as well as recognition of similarities between PECs and DECs.

Variables	1	2	3	4	5	6	7
1 Knowledge about eco-directed pharmaceutical disposal	1						
2 Attitudes toward eco-directed pharmaceutical disposal	0.791**	1					
3 Practice regarding eco-directed pharmaceutical disposal	0.576**	0.599**	1				
4 Perceived similarity between PECs and DECs	0.607**	0.635**	0.586**	1			
5 Knowledge about pro-environmental disinfectant use	0.722**	0.639**	0.542**	0.621**	1		
6 Attitudes toward pro-environmental disinfectant use	0.708**	0.807**	0.567**	0.613**	0.753**	1	
7 Practice regarding pro-environmental disinfectant use	0.444**	0.585**	0.598**	0.421**	0.451**	0.598**	1

Pearson correlation analysis was used to examine the relationships between the studied variables. Values presented in the table correspond to Pearson’s correlation coefficients (r); ** indicates that the correlation is significant at the *p* < 0.01 level (two-tailed test).

### 3.3 Regression analysis for the spillover effect

The regression equation Y = β_0_ + β_1_X_1_ + β_2_X_2_ + β_3_X_3_ + β_4_X_4_ + β_5_X_5_ + β_6_X_6_ + *ε* was used to test the spillover effects of eco-directed pharmaceutical disposal on pro-environmental disinfectant use across the KAP dimensions, respectively. Variables Y represented the pro-environmental disinfectant use score for one KAP dimensions, and the main explanatory variable X_1_ represented corresponding KAP score of eco-directed pharmaceutical disposal. Variables X_2_ to X_6_ were socio-demographic variables, including gender, age, place of residence, education level, and healthcare professional background. While β_0_ was a constant intercept, the *ε* value was the error.

Regression analysis for the spillover effect test (Table 3) showed that, when covariates were under control, an increase of one unit in eco-directed pharmaceutical disposal knowledge corresponded to an increase of 0.648 (*p* = 0.000) in knowledge regarding pro-environmental disinfectant use, thus the H1 was verified. For every one unit increase in the attitude toward eco-directed pharmaceutical disposal, the attitude score of pro-environmental disinfectant use increased by 0.782 points (*p* = 0.000), suggesting that the H2 was supported. In the practice dimension, a one-unit increase in eco-directed pharmaceutical disposal behaviors improved the residents’ pro-environmental disinfectant use behaviors by over 0.79 units (*p* = 0.000), which supported the H3.

**Table 3:** Regression analysis results of direct effects.

Variables	Knowledge about pro-environmental disinfectant use	Attitudes about pro-environmental disinfectant use	Practice regarding pro-environmental disinfectant use
Gender	0.007	0.037	−0.157***
Age	0.050*	0.003	−0.109***
Place of residence	−0.040*	0.001	0.087**
Education level	0.040*	0.031*	0.118***
Healthcare professional background	−0.072*	−0.016	0.224***
Knowledge about eco-directed pharmaceutical disposal	0.648***		
Attitudes toward eco-directed pharmaceutical disposal		0.782***	
Practice regarding eco-directed pharmaceutical disposal			0.791***
R	0.731	0.809	0.638
adj. R^2^	0.531	0.652	0.403
F	189.920	313.320	113.594

Regression analysis was used to test the direct effects of eco-directed pharmaceutical disposal on pro-environmental disinfectant use across the KAP dimensions, respectively. F, F statistic; R, coefficient of determination; adj. R^2^, adjusted coefficient of determination (squared). Other values presented in the table correspond to regression coefficients; *, **, and ** indicate that the direct effect is significant at the *p* < 0.05, *p* < 0.01, and *p* < 0.001 level (two-tailed test), respectively.

### 3.4 Test of the mediation effect

To test the H4, the bias-corrected non-parametric percentile Bootstrap method with the number of repeated samplings of 5,000 was employed to examine whether perceived similarity between PECs and DECs mediated the spillover effects of eco-directed pharmaceutical disposal on pro-environmental disinfectant use. Analyses were conducted separately for each KAP dimension, with the control of socio-demographic characteristics.

**Figure 1:**
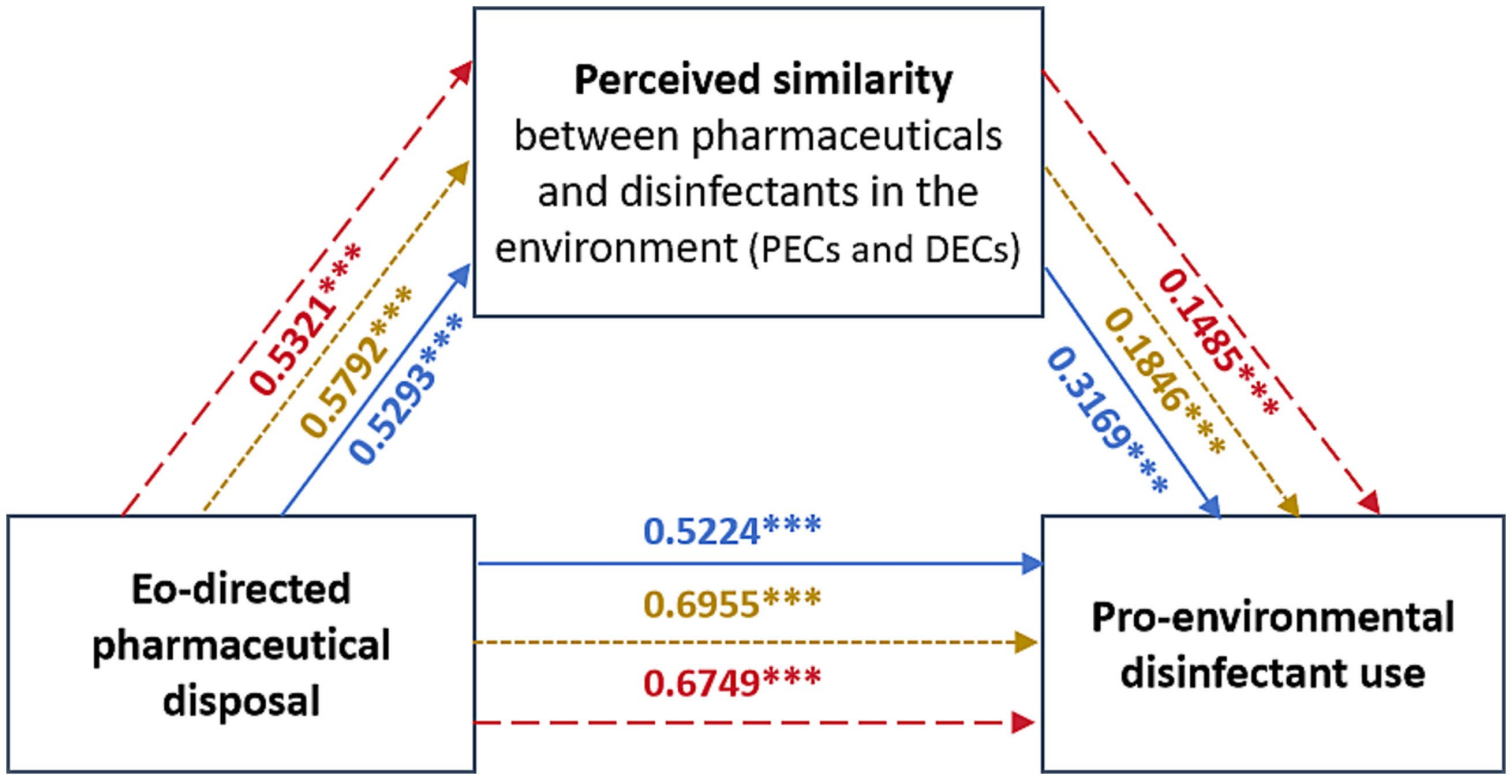
Model of the mediated role of the perceived similarity between pharmaceuticals and disinfectants in the environment (PECs and DECs) in the relationship between eco-directed pharmaceutical disposal and pro-environmental disinfectant use. Blue: knowledge dimension; Yellow: attitude dimension; Red: practice dimension. ***p < 0.001.

As shown in Table 4, the spillover effects of eco-directed pharmaceutical disposal on pro-environmental disinfectant use were significant in three studied dimensions, including knowledge (*β* = 0.691, *t* = 33.033, *p* = 0.000), attitude (*β* = 0.802, *t* = 43.152, *p* = 0.000), and practice (*β* = 0.754, *t* = 23.606, *p* = 0.000). Upon introducing the perceived similarity as the mediating variable, results showed significant indirect effects of eco-directed pharmaceutical disposal on pro-environmental disinfectant use *via* the perceived similarity, evidenced by an effect size value of 0.168 and a 95% confidence interval (CI) [0.130, 0.206] in K dimension, an effect size of 0.107 and a CI [0.072, 0.142] in A dimension, an effect size of 0.079 and a CI [0.029, 0.128] in P dimension, which accounted for 24.30, 13.31 and 10.48% of the total effects in the KAP dimensions, respectively. After controlling for the perceived similarity, the direct impacts of eco-directed pharmaceutical disposal on pro-environmental disinfectant use remained significant. The Lower Levels of the Bootstrap 95% confidence interval (LLCIs) were 0.474 (K), 0.694 (A), and 0.598 (P), the Upper Levels of Confidence Interval (ULCIs) were 0.517 (K), 0.742 (A), and 0.752 (P), respectively, and 0 was not included in the above confidence intervals. Figure 1 showed that all the path coefficients in the mediation model were significant. Collectively, these results confirmed that perceived similarity partially mediated the spillover effects of eco-directed pharmaceutical disposal on pro-environmental disinfectant use across KAP dimensions. Thus, the H4 was verified.

**Table 4:** Bootstrap mediating effects of perceived similarity.

Dimension	Effective	Effective size	BootSE	BootLLCI	BootULCI	Relative effective size %
Knowledge	Total effect	0.6901	0.0209	0.6491	0.7311	
	Direct effect	0.5224	0.0248	0.4737	0.5711	75.70
	Indirect effect	0.1677	0.0192	0.1298	0.2052	24.30
Attitude	Total effect	0.8024	0.0186	0.7659	0.8389	
	Direct effect	0.6955	0.0235	0.6494	0.7416	86.68
	Indirect effect	0.1068	0.0176	0.0720	0.1421	13.31
Practice	Total effect	0.7539	0.0319	0.6912	0.8165	
	Direct effect	0.6749	0.0392	0.5980	0.7518	89.52
	Indirect effect	0.0790	0.0256	0.0288	0.1281	10.48

## 4 Discussion

### 4.1 Eco-directed pharmaceutical disposal

Improper disposal of unwanted or expired pharmaceutical products stored in households is widely recognized as a critical contributor to PECs in the environment (14, 26, 29). A considerable number of residents are accustomed to casually disposing of their unwanted medications in trash, sinks and toilets (14, 43). Large quantities of unwanted household medications discarded in trash ultimately end up in municipal solid waste landfills. However, in developing countries including China, where solid waste treatment processes mainly rely on incineration, active pharmaceutical ingredients in landfills would gradually percolate into leachate and eventually be released into groundwater and other surrounding environmental matrices (44). In addition, as a representative class of emerging contaminants, pharmaceuticals that are discharged into municipal sewage systems after being discarded in sinks and toilets cannot be effectively and adequately removed in the activated sludge sewage treatment plants currently used in China (27, 30).

As a well-accepted eco-directed approach for pharmaceutical waste disposal, drug take-back systems enable the public to return unwanted household medications to pharmacies and health centers. These returned medications then undergo harmless treatment, thereby reducing the adverse environmental impacts posed by PECs (30, 39, 40, 43). A recent review of global academic research about drug take-back programs revealed that the environmental benefits associated with such programs have been gradually recognized by the public (43). Empirical evidence from Europe (45) and China (40) collectively demonstrated that providing information about the environmental hazards caused by improper disposal of unwanted drugs significantly increased the likelihood of returning household pharmaceutical waste to drug take-back systems among the residents. Consistent with the previous findings (40, 43, 45), our survey results derived from questions regarding respondents’ compliance with eco-directed pharmaceutical disposal practices (Q13-16) showed that most Chinese residents are concerned about the adverse environmental impacts of unwanted or expired medications, and tend to actively engage in eco-directed pharmaceutical disposal, with an average score of 3.83 out of 5.

EPV has a theoretical and practical foundation spanning over two decades in global leaders such as the European Union and the United States (26, 28), however, its implementation remains in infancy in developing countries represented by China. Nevertheless, in recent years, China has strengthened its focus on pharmaceutical environmental risks. Pharmaceuticals have been officially recognized as an important class of emerging contaminants requiring targeted attention, and antibiotics were formally included in the China’s national list of priority emerging contaminants in 2023 (39). As a core EPV measure and eco-friendly avenue for disposing of expired or unused medications, the drug take-back system has undergone vigorous development across China (30, 39, 40). A previous study has shown that an EPV intervention integrating multiple measures, including the take-back collection of unused drugs, effectively reduced PEC pollution in the rural water environment of China (30). Therefore, this study selected eco-directed pharmaceutical disposal (i.e., participation in the drug take-back system) as the initial PEB to investigate its spillover to pro-environmental disinfectant use.

### 4.2 Pro-environmental disinfectant use

Compared to eco-directed pharmaceutical disposal, that is already being steadily implemented in China, environmental issues associated with disinfectant disposal remain an emerging area of academic focus (4, 10, 12), and might be completely unfamiliar to most Chinese residents. To the best of our knowledge, the lack of laws or programs for the proper disposal of disinfectants is not unique to China, but rather such frameworks remain absent globally. However, with the growth of China’s green product market, the replacement of traditional disinfectants with environmentally friendly alternatives (e.g., natural disinfectants, non-chlorine oxidizing disinfectants, and nano-disinfectants) has gained recent attention (1, 12, 41). Currently, environmentally friendly disinfection products containing natural antimicrobial ingredients are commercially available on the Chinese market. A previous survey conducted among 1,861 Chinese residents has shown that the respondents had a certain understanding of environmentally friendly disinfectants and held very positive attitudes toward their development, consumption and application (41). Moreover, although the number is not large, a certain proportion of respondents (approximately 10–16%) were using environmental friendly disinfectants in their households during the survey period (41). Importantly, the adoption of green alternatives is also one of the core EPV measures (26, 29, 30). Therefore, the practice of using environmentally friendly disinfectants as green alternatives was focused on in the present study as the PEB related to disinfectant use.

### 4.3 Spillover of eco-directed pharmaceutical disposal on pro-environmental disinfectant use

The concept of environmental spillover has garnered growing attention in the field of environmental psychology (35, 36, 46). Positive environmental spillover occurs when the performance of an initial PEB increases the probability of performing a subsequent PEB. For instance, engaging in recycling positively spills over to organic product purchasing (35), and water conservation has a positive spillover effect on intention to save electrical energy (47). Importantly, when the positive environmental spillover exists, a prompting intervention targeting one specific behavior would subsequently promote a different and non-targeted behavior, thereby increasing the amount of PEBs that individuals perform (35, 37, 38). A review paper summarizing recent environmental spillover research (35) identified a critical gap, that is a narrow focus on limited sets of PEB exhibited in existing studies, with consumerism, energy conservation, recycling, and waste sorting being the most frequently investigated. While the range of examined behaviors needs further expansion, and diverse contextual settings should be integrated into future research. To our knowledge, the present study is the first to examine the potential spillover link between two healthcare-related pro-environmental behaviors from an environmental psychology perspective.

In China, disinfectants and pharmaceuticals are under supervision as two distinct product categories. Disinfection and infection control fall under the jurisdiction of disease control and sanitation management institutions, which operate under the National Health Commission (48). The production, use, and sale of disinfectant products are primarily governed by the Law on the Prevention and Treatment of Infectious Diseases. While the pharmaceutical products have a dedicated regulatory system and are mainly regulated by the Pharmaceutical Affairs Law under the leadership of the National Medical Products Administration (49, 50). Despite such regulatory gaps, this study taking 1,002 Chinese resident samples as examples indicated that good understanding, proactive attitudes and practical behavior of eco-directed pharmaceutical-related measures under the EPV principle might have positive impacts on the tendency and likelihood of engaging in pro-environmental disinfectant use. These findings thus supplemented empirical evidence for the feasibility of adopting EPV-related drug administration measures to constrain the environmental discharge of DECs from the anthropogenic source.

The spillover effects of eco-directed pharmaceutical disposal on pro-environmental disinfectant use across KAP dimensions of the residents, which were identified in this study, align with a previous pilot study (12) showing that a small sample (several dozen) of participants with EPV experience exhibited significantly stronger awareness of and more positive attitudes toward DEC-related environmental risks and their source control. These converging findings suggest that the implementation and promotion of EPV measures represented by eco-directed pharmaceutical disposal could not only control the anthropogenic emission of pharmaceuticals to the environment, but also produce a greater return, which is the remediation of DECs *via* improving residents’ pro-environmental disinfectant use, by triggering positive spillover.

### 4.4 Mediation effect of perceived similarity

To verify the previously proposed hypothesis that EPV can be adapted to reduce risks of DECs, based on shared characteristics between PECs and DECs (4, 10, 12), this study focused on perceived similarity as antecedents of environmental spillovers. The similarity between pairs of PEB has been considered enough to reinforce the positive spillover effect as a moderating mechanism (31–33, 35–38, 51). Thᴓgersen and Olander (51) proposed that PEBs within the same behavioral category tended to be more strongly correlated than those across different behavioral categories. Conceptually, the likelihood of pro-environmental conduct spreading from one behavior to another relies on how closely these two behaviors are associated in the person’s mind, while the association increases with their perceived similarity. A recent review (35) showed that, of six included studies that evaluated behavioral similarity as a potential spillover predictor, 100% verified its significance as a robust factor promoting spillover effects, underscoring the significant relevance of participants’ perception of task similarity in influencing spillover effects. Our study showed that most respondents recognized similarities between PECs and DECs, evidenced by an average perceived similarity score of 4.43 (out of 5). Consistent with previous studies (31–33, 35–38, 51), our mediation effect test validated that perceived similarity exerted an indirect promotional effect (partial mediation) on the relationship between eco-directed pharmaceutical disposal and pro-environmental disinfectant use across all KAP dimensions. This mediating role implies that implementing EPV measures could simultaneously improve residents’ pro-environmental disinfectant use by strengthening their recognition of PEC-DEC similarities. Therefore, targeted publicity and public education initiatives can be developed to highlight the shared characteristics of PECs and DECs, such as similar environmental risks as emerging contaminants, shared anthropogenic emission sources, analogous environmental pathways, comparable emission control strategies, and overlapping regulatory goals (4, 10, 12). By reinforcing these commonalities, such strategies can enhance perceived similarity among residents, thereby amplifying the positive spillover effects between EPV practices (e.g., drug take-back) and pro-environmental disinfectant use.

### 4.5 Limitations

This pilot study had some limitations. First, the Chinese residents included in this study were recruited through the snowball procedure, with the initial pool consisting of a convenience sample of university-enrolled graduate students. This is not a random sampling approach, which might affect the representativeness of the sample. Second, whether EPV measures other than eco-directed pharmaceutical disposal, such as the use of green alternatives or sustainable prescribing, also spill over to the residents’ pro-environmental disinfectant use is not addressed in this study. Third, we explored the spillover of eco-directed pharmaceutical disposal on pro-environmental disinfectant use based on cross-sectional survey data. Further empirical results are needed to understand the practical application of spillover dynamics. Moreover, because the original postulation that EPV could be adopted to reduce environmental risks posed by disinfectant emerging contaminants was previously proposed mainly based on the possible PEC-DEC similarities (4, 10, 12), this study focused on perceived similarity as a single mediating variable. However, other mediating variables still exist, such as environmental consciousness, pro-environmental self-identity, and concern. Future research should explore a more comprehensive set of mediating pathways to refine understanding of the mechanisms linking EPV-related behaviors to and pro-environmental disinfectant use.

## 5 Conclusion

Using survey data from 1,002 Chinese residents, this study provided initial empirical evidence of positive spillovers between eco-directed pharmaceutical disposal and pro-environmental disinfectant use across the KAP dimensions. Notably, residents’ perceived similarity between PECs and DECs exerted a partial mediating effect on these spillover relationships. Findings offer additional empirical support for the start-up of adopting EPV to combat the environmental issues posed by DECs. Therefore, despite the need for specific laws and regulations to prevent and control disinfectant pollution, it might be feasible to apply certain EPV measures, such as environmental education actions, take-back collection, and reverse logistics, in the risk control for DECs. Greater dissemination of the importance of EPV can help the public strengthen their environmental consciousness and improve their capabilities for addressing emerging contaminants including DECs.

## Data Availability

The raw data supporting the conclusions of this article will be made available by the authors, without undue reservation.
