# Supplementary material for: Potential spillover effect of eco-directed pharmaceutical disposal on pro-environmental disinfectant use among Chinese residents
Source: Front Public Health. 2025 Nov 26;13:1707924. doi: 10.3389/fpubh.2025.1707924 (PMC12689939; doi:10.3389/fpubh.2025.1707924)
Supplement: Supplementary file 1 [file Table_1.DOCX]

Supplementary Material

***TRANSLATED QUESTIONNAIRE***

**1- Socio-demographic characteristics.**

**Q1: Gender**(single choice) ○ Male ○ Female

**Q2: Years of age**(single choice) ○ < 18 ○ 18-20 ○ 21-40 ○ 41-65 ○ >65

**Q3: Place of residence** (single choice)

○ Municipalities or provincial capital cities

○ Other cities

○ Villages and towns

**Q4: Education level** (single choice)

○ High school or below ○ Junior college

○ Undergraduate ○ Postgraduate

**Q5: Healthcare professional background** (single choice)

○ Yes ○ No

***Please specify whether you agree or disagree with the following statements (Q6-31):***

**2- Eco-directed pharmaceutical disposal.**

***2.1 Knowledge dimension.***

**Q6: Pharmaceuticals used in healthcare practices would finally enter into the environment.** (single choice)

○ Strongly disagree. ○ Disagree. ○ Neutral. ○ Agree. ○ Strongly agree.

**Q7: Pharmaceutical residues in environment could cause adverse effects on the ecosystem.** (single choice)

○ Strongly disagree. ○ Disagree. ○ Neutral. ○ Agree. ○ Strongly agree.

**Q8: Improper disposal of unused/expired pharmaceutical products would result in pharmaceutical pollution in the environment.** (single choice)

○ Strongly disagree. ○ Disagree. ○ Neutral. ○ Agree. ○ Strongly agree.

***2.2 Attitude dimension.***

**Q10: Pharmaceuticals in the environment as a class of contaminants made me very worried, because they would pose adverse impacts on human health and the ecosystem.** (single choice)

○ Strongly disagree. ○ Disagree. ○ Neutral. ○ Agree. ○ Strongly agree.

**Q11: It is necessary to minimize the entrance of pharmaceuticals into the environment and its environmental risks.** (single choice)

○ Strongly disagree. ○ Disagree. ○ Neutral. ○ Agree. ○ Strongly agree.

**Q12: If there is an upstream intervention for controlling pharmaceutical entry**

**to the environment, I would endorse it, and be very pleased to participate in its**

**implementation.** (single choice)

○ Strongly disagree. ○ Disagree. ○ Neutral. ○ Agree. ○ Strongly agree.

***2.3 Practice dimension.***

**Q13: When disposing unwanted or expired pharmaceutical products, I am concerned about their possible adverse environmental impacts.** (single choice)

○ Strongly disagree. ○ Disagree. ○ Neutral. ○ Agree. ○ Strongly agree.

**Q14: Usually, I actively acquire knowledge about the environmental pollution caused by pharmaceuticals.** (single choice)

○ Strongly disagree. ○ Disagree. ○ Neutral. ○ Agree. ○ Strongly agree.

**Q15: I always take medications by the order of the validity, because I am concerned about the adverse environmental impacts of expired pharmaceuticals.** (single choice)

○ Strongly disagree. ○ Disagree. ○ Neutral. ○ Agree. ○ Strongly agree.

**Q16: In reality, I tend to return unwanted or expired pharmaceutical products to the take-back system in pharmacies, communities, or hospitals.**(single choice)

○ Strongly disagree. ○ Disagree. ○ Neutral. ○ Agree. ○ Strongly agree.

**3- Pro-environmental disinfectant use.**

***3.1 Knowledge dimension.***

**Q17: Disinfectants used in healthcare practices would finally enter into the environment.** (single choice)

○ Strongly disagree. ○ Disagree. ○ Neutral. ○ Agree. ○ Strongly agree.

**Q18: Disinfectant residues in environment could cause adverse effects on the ecosystem.** (single choice)

○ Strongly disagree. ○ Disagree. ○ Neutral. ○ Agree. ○ Strongly agree.

**Q19: Compared with traditional chemical disinfectants, natural disinfectants derived from herbs, microorganisms, *etc*. are more environmentally friendly.** (single choice)

○ Strongly disagree. ○ Disagree. ○ Neutral. ○ Agree. ○ Strongly agree.

***3.2 Attitude dimension.***

**Q20: Disinfectants in the environment as a class of contaminants made me very worried, because they would pose adverse impacts on human health and the ecosystem.** (single choice)

○ Strongly disagree. ○ Disagree. ○ Neutral. ○ Agree. ○ Strongly agree.

**Q21: It is necessary to minimize the entrance of disinfectants into the environment and its environmental risks.** (single choice)

○ Strongly disagree. ○ Disagree. ○ Neutral. ○ Agree. ○ Strongly agree.

**Q22: If there is an upstream intervention for controlling disinfectant entry**

**to the environment, I would endorse it, and be very pleased to participate in its**

**implementation.** (single choice)

○ Strongly disagree. ○ Disagree. ○ Neutral. ○ Agree. ○ Strongly agree.

***3.3 Practice dimension.***

**Q23: When choosing disinfectant products, I am concerned about their possible adverse environmental impacts.** (single choice)

○ Strongly disagree. ○ Disagree. ○ Neutral. ○ Agree. ○ Strongly agree.

**Q24: Usually, I actively acquire knowledge about the environmental pollution caused by disinfectants.** (single choice)

○ Strongly disagree. ○ Disagree. ○ Neutral. ○ Agree. ○ Strongly agree.

**Q25: In reality, I tend to use environmentally friendly disinfectants for hand sanitization or household environmental disinfection, because I am concerned about the adverse environmental impacts of disinfectants.** (single choice)

○ Strongly disagree. ○ Disagree. ○ Neutral. ○ Agree. ○ Strongly agree.

**Q26: In reality, I tend to consume and use environmentally friendly disinfectant products, even if their prices are sometimes higher.**(single choice)

○ Strongly disagree. ○ Disagree. ○ Neutral. ○ Agree. ○ Strongly agree.

**4- Judgments about the possible similarities between disinfectants and pharmaceuticals in the environment.**

**Q26:** : **Both disinfectant residues and pharmaceutical residues in environment are emerging contaminants. No legal control over their environmental issues has been set up in China.**(single choice)

○ Strongly disagree. ○ Disagree. ○ Neutral. ○ Agree. ○ Strongly agree.

**Q27:** : **Both disinfectants and pharmaceuticals in the environment could cause adverse effects on ecosystem and wildlife species.**(single choice)

○ Strongly disagree. ○ Disagree. ○ Neutral. ○ Agree. ○ Strongly agree.

**Q28:** : **Both disinfectants and pharmaceuticals in the environment could cause the emergence of drug-resistant bacteria.**(single choice)

○ Strongly disagree. ○ Disagree. ○ Neutral. ○ Agree. ○ Strongly agree.

**Q29:** : **The sources of both disinfectant pollution and pharmaceutical pollution in environment could be traced back to the healthcare behaviors.**(single choice)

○ Strongly disagree. ○ Disagree. ○ Neutral. ○ Agree. ○ Strongly agree.

**Q30:** : **The possible entrance routes of disinfectants and pharmaceuticals into the environment are similar.**(single choice)

○ Strongly disagree. ○ Disagree. ○ Neutral. ○ Agree. ○ Strongly agree.

**Q31: As both disinfectants and pharmaceuticals are managed by the health administrative departments, these two kinds of environmental pollution might be controlled by the same regulation system.**(single choice)

○ Strongly disagree. ○ Disagree. ○ Neutral. ○ Agree. ○ Strongly agree.
